# Supplementary material for: A multi-protein receptor-ligand complex underlies combinatorial dendrite guidance choices in C. elegans
Source: eLife. 2016 Oct 5;5:e18345. doi: 10.7554/eLife.18345 (PMC5079751; doi:10.7554/eLife.18345)
Supplement: Supplementary file 1. — (a) Strains used in this study. (b) Plasmids used in this study. DOI: http://dx.doi.org/10.7554/eLife.18345.020 [file elife-18345-supp1.docx]

**Supplemental file 1a. Strains used in this study**

| Strain name | Detailed information | Notes |
| --- | --- | --- |
| TV20654 | *wyIs592 [ser2prom3>myr-gfp] wyEx8500 [Pnhr-81>mcherry, Pmyo-3>mcherry and Pmyo-2>mcherry]* | Used in Fig 1-7 and Figure 6-figure supplement 1, to image and quantify the PVD dendrites in WT |
| TV20826 | *lect-2(wy953); wyIs592 [ser2prom3>myr-gfp] wyEx8500 [Pnhr-81>mcherry, Pmyo-3>mcherry and Pmyo-2>mcherry]* | Used in Fig 1 and 3, to image and quantify the PVD dendrites in *wy953* mutants |
| TV20655 | *lect-2(ok2617); wyIs592 [ser2prom3>myr-gfp] wyEx8500 [Pnhr-81>mcherry, Pmyo-3>mcherry and Pmyo-2>mcherry]* | Used in Fig 2, 5, 6, 7 and Figure 6-figure supplement 1, to image and quantify the PVD dendrites in *ok2617* mutants |
| TV20626 | *lect-2(ok2617); wyTi3 [loxP-Plect-2>lect-2 genomic:: SL2 ::mCHERRY :: unc-54 3’UTR -loxP]; wyIs592 [ser2prom3>myr-gfp]* | Used in Fig 2 and 5, to show that *wyTi3* fully rescue the PVD defects of *ok2617* mutants |
| TV20828 | *sax-7(nj48); wyIs592 [ser2prom3>myr-gfp] wyEx8500 [Pnhr-81>mcherry, Pmyo-3>mcherry and Pmyo-2>mcherry]* | Used in Fig 3, to image and quantify the PVD dendrites in *sax-7* single mutants |
| TV20829 | *mnr-1(wy758); wyIs592 [ser2prom3>myr-gfp] wyEx8500 [Pnhr-81>mcherry, Pmyo-3>mcherry and Pmyo-2>mcherry]* | Used in Fig 3, to image and quantify the PVD dendrites in *mnr-1* single mutants |
| TV20844 | *dma-1(wy686); wyIs592 [ser2prom3>myr-gfp] wyEx8500 [Pnhr-81>mcherry, Pmyo-3>mcherry and Pmyo-2>mcherry]* | Used in Fig 3, to image and quantify the PVD dendrites in *dma-1* single mutants |
| TV20855 | *lect-2(wy953); sax-7(nj48); wyIs592 [ser2prom3>myr-gfp] wyEx8500 [Pnhr-81>mcherry, Pmyo-3>mcherry and Pmyo-2>mcherry]* | Used in Fig 3, to image and quantify the PVD dendrites in *lect-2; sax-7* double mutants |
| TV20856 | *lect-2(wy953); mnr-1(wy758); wyIs592 [ser2prom3>myr-gfp] wyEx8500 [Pnhr-81>mcherry, Pmyo-3>mcherry and Pmyo-2>mcherry]* | Used in Fig 3, to image and quantify the PVD dendrites in *lect-2; mnr-1* double mutants |
| TV20854 | *dma-1(wy686); lect-2(wy953); wyIs592 [ser2prom3>myr-gfp] wyEx8500 [Pnhr-81>mcherry, Pmyo-3>mcherry and Pmyo-2>mcherry]* | Used in Fig 3, to image and quantify the PVD dendrites in *dma-1;* *lect-2*double mutants |
| TV20625 | *lect-2(ok2617); unc-119(ed4); wyTi3 [loxP-Plect-2>lect-2 genomic:: SL2 ::mCHERRY :: unc-54 3’UTR -loxP]* | Used in Fig 4 to determine the transcriptional expression pattern of *lect-2* |
| TV20834 | *lect-2(ok2617); wyIs592; wyTi3 [loxP-Plect-2>lect-2 genomic:: SL2 ::mCHERRY :: unc-54 3’UTR -loxP] wyEx8554 [Phlh-1>wormCre, Podr-1>gfp and Pmyo-2>mcherry]* | Used in Fig 5, to make muscle conditional knockouts |
| TV20835 | *lect-2(ok2617); wyIs592; wyTi3 [loxP-Plect-2>lect-2 genomic:: SL2 ::mCHERRY :: unc-54 3’UTR -loxP] wyEx8555 [Phlh-1>wormCre, Podr-1>gfp and Pmyo-2>mcherry]* | Used in Fig 5, to make muscle conditional knockouts |
| TV20839 | *lect-2(ok2617); wyIs592 [ser2prom3>myr-gfp] wyEx8559 [Phlh-1>lect-2::sl2::mcherry, Pnhr-81>mcherry, Pmyo-2> mcherry and Podr-1>gfp ]* | Used in Fig 6 to do muscle mosaic analysis |
| TV20786 | *lect-2(ok2617); wyIs592 [ser2prom3>myr-gfp] wyEx8549 [Phlh-1>lect-2::sl2::mcherry, Pnhr-81>mcherry, Pmyo-2> mcherry and Podr-1>gfp ]* | Used in Fig 7 and Fig 6-figure supplement 1, to do muscle specific rescue |
| TV20786 | *lect-2(ok2617); wyIs592 [ser2prom3>myr-gfp] wyEx8550 [Phlh-1>lect-2::sl2::mcherry, Pnhr-81>mcherry, Pmyo-2> mcherry and Podr-1>gfp ]* | Used in Fig 6-figure supplement 1, to do muscle specific rescue |
| TV20627 | *lect-2(ok2617); wyIs592 [ser2prom3>myr-gfp] wyEx8490*  *[Pnhr-81>lect-2::sl2::mcherry, Pnhr-81>mcherry, Pmyo-2> mcherry, Pmyo-3>mcherry and Podr-1>gfp ]* | Used in Fig 7 and Fig 6-figure supplement 1, to do seam cell specific rescue |
| TV20628 | *lect-2(ok2617); wyIs592 [ser2prom3>myr-gfp] wyEx8490*  *[Pnhr-81>lect-2::sl2::mcherry, Pnhr-81>mcherry, Pmyo-2> mcherry, Pmyo-3>mcherry and Podr-1>gfp ]* | Used in Fig 7 and Fig 6-figure supplement 1, to do seam cell specific rescue |
| TV19699 | *lect-2(ok2617); wyIs592 [ser2prom3>myr-gfp] wyEx8131*  *[ser2prom3>lect-2, Pmyo-2>mcherry and Punc-122>rfp]* | Used in Fig 6-figure supplement 1, to do PVD specific rescue |
| TV19700 | *lect-2(ok2617); wyIs592 [ser2prom3>myr-gfp] wyEx8132*  *[ser2prom3>lect-2, Pmyo-2>mcherry and Punc-122>rfp]* | Used in Fig 6-figure supplement 1, to do PVD specific rescue |
| TV19702 | *lect-2(ok2617); wyIs592 [ser2prom3>myr-gfp] wyEx8134*  *[Pdpy-7>lect-2, Pmyo-2>mcherry and Punc-122>rfp]* | Used in Fig 6-figure supplement 1, to do epidermis specific rescue |
| TV19703 | *lect-2(ok2617); wyIs592 [ser2prom3>myr-gfp] wyEx8135*  *[Pdpy-7>lect-2, Pmyo-2>mcherry and Punc-122>rfp]* | Used in Fig 6-figure supplement 1, to do epidermis specific rescue |
| TV20306 | *lect-2(ok2617); wyIs592 [ser2prom3>myr-gfp] wyEx8341*  *[Pmyo-2>lect-2, Pmyo-3>mcherry and Punc-122>rfp]* | Used in Fig 6-figure supplement 1, to do pharyngeal muscle specific rescue |
| TV20307 | *lect-2(ok2617); wyIs592 [ser2prom3>myr-gfp] wyEx8341*  *[Pmyo-2>lect-2, Pmyo-3>mcherry and Punc-122>rfp]* | Used in Fig 6-figure supplement 1, to do pharyngeal muscle specific rescue |
| TV20832 | *lect-2(wy953); sax-7(nj48); wyIs592 [ser2prom3>myr-gfp] wyEx8490*  *[Pnhr-81>lect-2::sl2::mcherry, Pnhr-81>mcherry, Pmyo-2> mcherry, Pmyo-3>mcherry and Podr-1>gfp ]* | Used in Fig 7, to determine whether *sax-7* is required for seam cell targeting induced by *seam cell>lect-2* |
| TV20833 | *lect-2(wy953); mnr-1(wy758); wyIs592 [ser2prom3>myr-gfp] wyEx8490*  *[Pnhr-81>lect-2::sl2::mcherry, Pnhr-81>mcherry, Pmyo-2> mcherry, Pmyo-3>mcherry and Podr-1>gfp ]* | Used in Fig 7, to determine whether *mnr-1* is required for seam cell targeting induced by *seam cell>lect-2* |
| TV20831 | *dma-1(wy686); lect-2(wy953); wyIs592 [ser2prom3>myr-gfp] wyEx8490*  *[Pnhr-81>lect-2::sl2::mcherry, Pnhr-81>mcherry, Pmyo-2> mcherry, Pmyo-3>mcherry and Podr-1>gfp ]* | Used in Fig 7, to determine whether *dma-1* is required for seam cell targeting induced by *seam cell>lect-2* |
| TV20148 | *unc-119(ed4) wyEx8272 [Plect-2>lect-2::gfp, Psax-7>sax-7::mcherry, unc-119(+)]* | Used in Fig 8, 9 and Fig 9-figure supplement 1, to test LECT-2-SAX-7 colocalization and do SiMPull |
| TV21337 | *dma-1[wy1041(dma-1::2xflag)]; unc-119(ed4) wyEx8272 [Plect-2>lect-2::gfp, Psax-7>sax-7::mcherry, unc-119(+)]* | Used in Fig 9 and Fig 9-figure supplement 1, to do SiMPull |
| TV21437 | *dma-1[wy1041(dma-1::2xflag)]; unc-119(ed4);mnr-1(wy758) wyEx8272 [Plect-2>lect-2::gfp, Psax-7>sax-7::mcherry, unc-119(+)]* | Used in Fig 9 and Fig 9-figure supplement 1, to do SiMPull |
| TV21309 | *lect-2[wy1090(yfp::lect-2::unc-119)]; unc-119(ed4)* | Used in Fig 4 and 8, to determine the expression pattern of endogenously expressed LECT-2 |
| TV21394 | *lect-2(wy1090(yfp::lect-2::unc-119)); wyIs592[ser2prom3>myr-gfp]* | Used in Fig 4, to determine whether the yfp insertion affects the function of endogenous *lect-2* |
| TV21675 | *lect-2[wy1090(yfp::lect-2::unc-119)];sax-7(nj48)* | Used in Fig 8, to determine whether the localization of YFP::LECT-2 is SAX-7-dependent |
| TV21676 | *lect-2[wy1090(yfp::lect-2::unc-119)];mnr-1(wy758)* | Used in Fig 8, to determine whether the localization of YFP::LECT-2 is MNR-1-dependent |
| TV17697 | *wyIs50001[Pdpy-7::sax-7::yfp and ser2prom3>myr-mcherry]* | Fig 1-figure supplement 1, to determine the relationship between the pattern of epidermis-expressed SAX-7 and PVD morphology |
| TV19629 | *unc-119(ed4) wyEx8083 [Plect-2>lect-2 genomic:: gfp:: SL2:: mcherry]* | Fig 8-figure supplement 1, to determine the transcriptional expression pattern of *lect-2* |
| TV20156 | *unc-119(ed4); sax-7(nj48)wyEx8083 [Plect-2>lect-2 genomic:: gfp:: SL2:: mcherry]* | Fig 8-figure supplement 1, to determine whether sax-7 affects the transcriptional expression pattern of *lect-2* |

**Supplemental file 1b. Plasmids used in this study**

| Plasmid name | Detailed information | Notes |
| --- | --- | --- |
| pWZ350 | *Pnhr-81>mcherry::unc-54 3’UTR* | To label seam cells |
| pCFJ104 | *Pmyo-3>mcherry::unc-54 3’UTR* | To label body wall muscles  (a gift from Dr. Erik Jorgensen) |
| pWZ338 | *miniMos-loxp-Plect-2 (1.5kb)>lect-2 genomic:: SL2 ::mcherry ::unc-54 3’UTR-loxp* | To generate *wyTi3* |
| pWZ344 | *Phlh-1> wormCre::tbb-2 UTR* | To express worm codon optimized Cre in body wall muscles |
| pWZ233 | *ser2prom3>lect-2cDNA::unc-54 3’utr* | To express *lect-2* in PVD neurons |
| pWZ234 | *Pdpy-7> lect-2cDNA::unc-54 3’utr* | To express *lect-2* in epidermis |
| pWZ265 | *Pmyo-2> lect-2cDNA::unc-54 3’utr* | To express *lect-2* in pharyngeal muscles |
| pWZ299 | *Phlh-1>lect-2 cDNA::SL2::mcherry::unc-54 3’UTR* | To express *lect-2* and *mcherry* in body wall muscles |
| pWZ300 | *Pnhr-81>lect-2 cDNA::SL2::mcherry::unc-54 3’UTR* | To express *lect-2* and *mcherry* in seam cells |
| pWZ272 | *Plect-2(3.4kb)>lect-2 genomic::gfp::unc-54 3’ UTR* | To determine the protein localization of LECT-2 |
| pWZ226 | *Plect-2(3.4kb)>lect-2 genomic::gfp::SL2::mcherry::unc-54 3’UTR* | To determine the protein localization of LECT-2 and its transcriptional pattern |
| pXD18 | *Psax-7>sax-7::mcherry::sax-7 3’ UTR (recombined fosmid)* | To determine the protein localization of SAX-7 |
| pWZ374 | *PU6>lect-2-sgRNA #1* | To generate *yfp::lect-2* knock-in strain |
| pWZ375 | *PU6>lect-2-sgRNA#2* | To generate *yfp::lect-2* knock-in strain |
| pJW1259 | *Peft-3>cas9* | To generate *yfp::lect-2* knock-in strain |
| pXD49 | *Pactin>sax-7::gfp* | To express SAX-7::GFP in *Drosophila* S2 cells |
| pXD53 | *Pactin>sax-7::3xHA* | To express SAX-7::3xHA in *Drosophila* S2 cells |
| pXD94 | *Pactin>sax-7 ECD::3xHA* | To express SAX-7 ECD::3xHA in *Drosophila* S2 cells (most of SAX-7 ECD is cleaved at a putative furin site in the third FnIII domain in S2 medium) |
| pWZ346 | *Pactin>sax-7 ECD (with furin site mutations)::3xHA* | To express SAX-7 ECD::3xHA in *Drosophila* S2 cells (resistant to cleavage in the third FnIII domain) |
| pWZ348 | *Pactin>lect-2::3xHA* | To express LECT-2::3xHA in *Drosophila* S2 cells |
| pWZ263 | *Pactin>lect-2::3xFLAG* | To express LECT-2::3xFLAG in *Drosophila* S2 cells |
| pXD85 | *Pactin>mnr-1::gfp* | To express MNR-1::GFP in *Drosophila* S2 cells |
| pXD54 | *Pactin>dma-1::rfp* | To express DMA-1::RFP in *Drosophila* S2 cells |
